# Corneal compensation of presbyopia: PresbyLASIK: an updated review

**DOI:** 10.1186/s40662-017-0075-9

**Published:** 2017-04-13

**Authors:** Veronica Vargas-Fragoso, Jorge L. Alió

**Affiliations:** 1 Vissum Corporation, Edificio Vissum, Calle Cabañal 1, Alicante, Spain; 2 Division of Ophthalmology, Universidad Miguel Hernández, Carretera Alicante-Valencia km 8.7, Alicante, Spain

**Keywords:** Central and peripheral PresbyLASIK, Corneal multifocality, Presbyopia

## Abstract

**Abstract:**

The main purpose of this review is to compare and analyze the results of the main PresbyLASIK approaches; central and peripheral.

**Summary:**

A comprehensive research was conducted in PUBMED using keywords like “presbyopia correction”, “PresbyLASIK”, “Corneal multifocality”, “Laser blended vision”. We reviewed the PresbyLASIK technique, uncorrected distance visual acuity (UDVA), corrected distance visual acuity (CDVA), uncorrected near visual acuity (UNVA), and corrected near visual acuity (CNVA), and compared the differences between the techniques.

## Background

Presbyopia is an age-related loss of accommodative amplitude; symptoms begin to appear after the age of 40. It is estimated that in 2050 there will be 1.782 billion people with presbyopia [1].

Its correction has always been challenging for the refractive surgeon. The static methods for its correction seek to increase the depth of focus, which include: monovision, corneal inlays, presbyLASIK, corneal shrinking techniques (conductive keratoplasty, laser thermal keratoplasty and intrastromal femtosecond laser-based procedures), multifocal IOLs [2]. The dynamic methods such as scleral implants and accommodative IOLs attempt to restore accommodation [2]. A corneal approach seems the safest, since it is the less invasive procedure.

Moreira et al. were the first ones to intentionally create a multifocal profile to correct myopia and maintain a good uncorrected near visual acuity (UNVA) by creating a central steeper area [3].

The term PresbyLASIK was introduced by Ruiz in 1996 [4]; it is a surgical technique based on the principles of LASIK to create a multifocal corneal surface.

There are 3 main types of multifocal corneal excimer laser profiles: 1) Multifocal transition profile, 2) Central PresbyLASIK, 3) Peripheral PresbyLASIK. The principles of each algorithm may be based on the dioptric power of refractive error and presbyopia correction calculation, corneal asphericity quotient (Q-value), higher-order spherical aberrations changes or optical and transition zone manipulation.

## Approaches

### Multifocal transition profile

This technique had some popularity in the 1980s; it created a transitional vertical multifocal ablation based on the creation of an intentional decentration of a hyperopic ablation profile. Gobien et al. reported an improvement of 1 line of near UCVA in hyperopic presbyopes [5]. There are very few reports on this technique and it was not well accepted by surgeons because it induced significant levels of vertical coma [6].

### Central PresbyLASIK

**Fig. 1:**
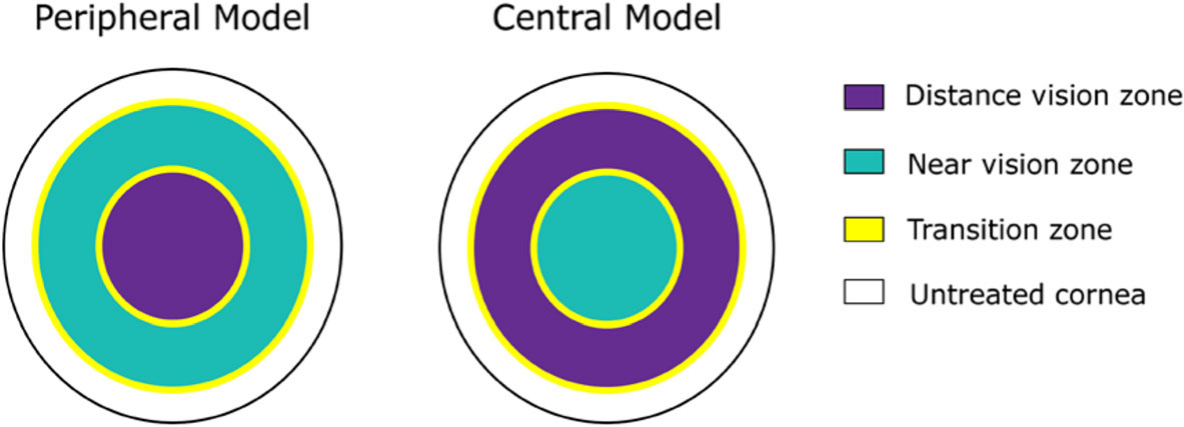
Differences between ablation patterns. In peripheral presbyLASIK, the center of the cornea is treated for distance vision and the periphery for near, while in central presbyLASIK, the center of the cornea is treated for near vision and the periphery for distance vision

This technique was first described by Ruiz in 1996 where it creates a hyperpositive area for the near vision at the center, and the periphery is left for far vision (Fig. 1). It is pupil-dependent and an advantage is that it can be performed at the center of the cornea in myopic and hyperopic profiles, and in emmetropes with minimal corneal excision. Adequate centration is crucial for having a controllable result. Its main limitation is the lack of adequate alignment among the line of sight, the central pupil and the corneal vertex, inducing coma aberrations.

### Peripheral PresbyLASIK

In this technique, the center of the cornea is left for distance and the periphery is ablated in a way that a negative peripheral asphericity is created to increase the depth of the field (Fig. 1). However, when positive spherical aberration is present and if the pupil becomes miotic, the refraction of the eye experiences a shift towards positive spherical values that negatively influences near-vision performance [6].

One of its disadvantages is that when it is used in association with myopic correction, it is necessary to remove a significant amount of corneal tissue and therefore is mainly performed in hyperopes. It also requires an efficient excimer laser beam profile that can compensate the loss of energy that happens while ablating the peripheral cornea; this is one of the main difficulties in specifically targeting high negative asphericity values with this technique. A relatively flatter central cornea and more highly curved corneal mid-periphery was described by Avalos (PARM technique), and a proprietary peripheral presbyLASIK algorithm was described and patented by Tamayo.

### Central PresbyLASIK technique

It is the most performed presbyLASIK technique [7], the first published results were reported by Alió et al. who reported a 6 months follow up in 25 hyperopes; 64% of the patients had an uncorrected distance visual acuity (UDVA) of 20/20, 72% of patients had a UNVA **>**20/40, and 28% of the patients had a loss of 2 lines of corrected distance visual acuity (CDVA). Coma aberrations increased and spherical aberrations decreased [8].

### AMO Visx hyperopia-presbyopia multifocal approach

(AMO Development LLC, Milpitas, California) This design steepens the central zone to improve near vision and the peripheral zone for distance vision. It is for hyperopes patients with up to +4.0 D and -2.00 D of astigmatism [9].

Jackson et al. [10] reported a 12 month follow up using an aspheric presbyopia treatment, and wavefront guided hyperopic LASIK treatment using the VISX STAR S4 excimer laser (AMO). Fifty eyes completed the 12-month follow up, 100% had a binocular uncorrected distance vision of 20/25 or better, and an uncorrected near vision of J3. Ten percent of patients had a loss of >2 lines of CDVA. Higher order aberrations increased after surgery, mainly negative spherical aberration, which was correlated with the improved near visual acuity.

### SUPRACOR

(Technolas Perfect Vision GmbH, Munich, Germany) is an aberration-optimized presbyopic algorithm. The Supracor creates a hyperpositive area in the central 3.0 mm zone (giving an addition of approximately 2 diopters [11]), the treatment targets 0.50 D of myopia in both eyes [12], being this the symmetrical technique, or it can be performed in an asymmetrical way, in which the target of the dominant eye is plano, and the non-dominant eye target is -0.50 D [4]. The asymmetrical technique is performed in patients that demand both near and distance vision, the symmetrical technique is for patients that demand good near vision [4]. It treats hyperopic presbyopia and minimizes the aberrations normally induced during treatment. This algorithm is available in the Teneo 317 and in the Technolas 217P excimer lasers [11] (Bausch and Lomb Technology, Munich Germany).

Ryan et al. reported the first results of the SUPRACOR technique. A binocular UDVA of 0.2 logMAR or better was achieved in 91% of the patients, also, 91% had an uncorrected reading ability of N8 or better, 6% lost 2 or more lines of CDVA, and 93% of the patients were fully independent of reading glasses. There was a small increase in higher order aberration (HOA) RMS, but no significant increase in coma or trefoil [12] .

A 1 year follow up by Schlote et al. [13] showed that 87.2% of the patients had an UNVA of >0.4 logMAR after Supracor, but 40% of the patients used reading glasses every day. Ten percent of the eyes lost 2 lines of CDVA.

Saib et al. reported a study using the SUPRACOR regular algorithm and a micro-monovision; 100% of the patients achieved a 20/25 distance vision and a 20/30 uncorrected near vision acuity 1 year after surgery. Eighty-four percent of patients achieved a simultaneous UDVA of 20/25 and UNVA of J1, 9.45% of patients lost one line of CDVA, and 4.05% lost 2 or 3 lines at 6 months. There was a more negative spherical aberration and vertical coma post operatively. Most of the patients (83.3%) were happy with their results [14].

Cosar et al. [15] performed a 6 months follow up, reporting an UNVA of 20/20 in 77.2% of eyes and 20/25 in 89.4% of the eyes, with a loss of 1 line in 28.5% of the eyes while 10.6% of the eyes lost 2 lines of CDVA.

### PresbyMAX

PresbyMAX (SCHWIND eye-tech-solutions GmbH, Kleinostheim, Germany) is based on the creation of a biaspheric multifocal corneal surface with a central hyper positive area to achieve +0.75 to +2.50 D of near vision correction, surrounded by an area in which the ablation is calculated to correct the distance refractive error [16, 17].

Uthoff et al. used a Presbymax approach in hyperopic, myopic and emmetropic patients; 83% of all patients had an UDVA of 0.1 logMAR or better (made up of 100% of hyperopic, 80% of emmetropic and 70% of myopic patients). Ninety percent of the emmetropic, and 80% of hyperopic and myopic eyes had an uncorrected near visual acuity (UCNVA) of 0.3 logRAD or better. Ten percent of the hyperopic patients lost 2 lines of best corrected distance visual acuity (BCDVA), and 40% lost 1 line, and the same was with the emmetropic group, while for myopic patients: 10% lost 3 lines, 10% lost 2 lines, and 10% lost 1 line of BCDVA. There was a shift into negative spherical aberration and neither third order trefoil nor coma were significantly changed postoperatively. The most satisfied group was the hyperopic group. There was no retreatment, although this was only a 6 months follow up study [18].

Luger et al. reported using PresbyMAX treatment in myopes and hyperopes with or without astigmatism and published the outcomes of a year follow up. Seventy percent of patients had UDVA of 0.1 logMAR or better, 84% had UNVA of 0.1 logRAD or better, and 85% of patients had UDVA of 0.2 logMAR and UNVA of 0.2 logRAD or better. Three percent of the eyes lost 2 lines of CDVA and 8% of the eyes lost 2 lines of corrected near visual acuity (CNVA) [19].

Baudu et al. analyzed the uncorrected binocular results of PresbyMAX at 6 months in myopic and hyperopic presbyopic patients. 76% of patients had a binocular UDVA of 0.1 logMAR or better, 91% had an UNVA of 0.1 logRAD or better. Eighty percent of the patients achieved binocular success (determined as UDVA of 0.15 logMAR or better and UNVA of 0.15 logRAD or better [17].

Luger et al. reported the outcomes of PresbyMAX and micro-monovision, in both myopic and hyperopic presbyopes 1 year postoperatively. The dominant eye had a target refraction of -0.1 D, and the non-dominant eye (near eye) a target refraction of -0.9 D. Ninety-three percent of patients had an UDVA of 20/20, 90% with UNVA of J2, 97% with uncorrected intermediate visual acuity (UIVA) of J2, and 7% lost 2 Snellen lines of CDVA [16].

Chan et al. reported a follow up of 1 year of combining PresbyMAX in the non-dominant eye and contralateral monofocal distance correction in the dominant eye, in patients with bilateral hyperopia and presbyopia. Eighty-seven percent of the patients had UDVA 20/25 or better, and 83% had UNVA Jaeger level J2 or better. Simultaneous binocular near and distance vision of 20/25 and J2 or better was achieved in 70% of the patients. No patient suffered from a loss of 2 Snellen lines of CDVA, and 14% of the patients had a retreatment to improve near vision within 6 months to 1 year postoperative. There was a statistically significant induction of negative spherical aberration after the procedure, and the change in total HOA was significantly different between fellow eyes. Ninety-four percent of the patients were satisfied with their visual outcome, 26% of patients reported difficulty in visual performance in a low illuminated environment [20].

See Table 1 for a review on the results of central PresbyLASIK.

**Table 1:** Published outcomes for presbyopia correction with Central PresbyLASIK

Author	Procedure	Follow up	No. of patients	Mean Age (years)	UNVA	UDVA	Safety	Spectacle independence / Satisfaction	Retreatments
Alió et al. [8]	Central PresbyLASIK	6 months	25 hyperopes	58	72% → >20/40	64% → 20/20	28% lost 2 lines of BSCVA	72% spectacle independence for all distances	12% standard LASIK for distance
Jackson et al. [10]	Central PresbyLASIK	12 months	25 hyperopes	55.1 ± 4.6	100% → J3	100% → 20/25	10% lost >2 lines of CDVA. 8.3% lost > 2 lines of CNVA, at 6 months.	80% did not use spectacles to write checks or for computer work. 40% could read without spectacles.	NA
Ryan et al. [12]	Supracor	6 months	23 hyperopes	57	91% → N8 or better	91% → 0.2 logMAR	6% lost 2 or more lines of CDVA	93% fully independent	22% retreatment to enhance UDVA
Saib et al. [14]	Supracor and micro-monovision	12 months	24 hyperopes	54.3 ± 4	84.21% → J1 94.73% → J2 or better	100% → 20/25	9.4% lost 1 line of CDVA 4.05% lost 2 or 3 lines of CDVA.	83.3% fully independent	6.75%
Uthoff et al. [18]	PresbyMAX	6 months	30 patients 10 myopic 10 emmetropic 10 hyperopic	54	0.3 logRAD or better 90% emmetropic 80% myopic and hyperopic	0.1logMAR → 100% hyperopic 80% emmetropic 70% myopic	10% lost 2 lines 40% lost 1 line of the hyperopic and emmetropic group. 10% myopes lost 3 lines, 10% lost 2 lines and 10% lost 1 line of BCDVA.	NA	None
Luger et al. [19]	PresbyMAX	1 year	31 patients myopic and hyperopic with or without astigmatism	53 ± 4	84% → 0.1 logRAD or better	70% → 0.1 logMAR or better	3% lost 2 lines of CDVA, 8% lost 2 lines of CNVA	NA	NA
Luger et al. [16]	PresbyMAX and micro-monovision	1 year	32 patients myopic and hyperopic	51 ± 3	90% → J2	93% → 20/20	7% lost 2 lines of CDVA	Improvement from little (preoperative) to high (postoperative) satisfaction	19%, to improve distance or near VA
Baudu et al. [17]	PresbyMAX	6 months	350 patients, myopes and hyperopes	53 ± 6	91% → 0.1 logRAD or better	76% → 0.1 logMAR or better	Myopes had a global loss averaged -0.8 ± 0.5 lines and ranged from -2 to 0 lines, hyperopes had a global loss averaged -0.9 ± 0.5 lines and ranged from -3 to 0 lines.	NA	NA
Chan et al. [20]	PresbyMAX in the non-dominant eye, monofocal LASIK in the dominant eye	1 year	36 hyperopes	53.1 ± 4	77% → J2 or better	87% → 20/25 or better	No patient had a loss of 2 Snellen lines of binocular CDVA.	94.4% of patients were satisfied with their outcomes	14% to improve near vision

*NA=* Information not available, *UNVA=* uncorrected near visual acuity, *UDVA=* uncorrected distance visual acuity, *BSCVA=* best spectacle-corrected acuity, *CNVA=* corrected near visual acuity, *CDVA=* corrected distance visual acuity, *BCDVA=* best corrected distance visual acuity

### Peripheral PresbyLASIK Technique

Peripheral Multifocal LASIK (PML) was described and developed by Pinelli; it creates a multifocal corneal profile in a 6.5 mm diameter zone. The distance correction is done at a 6 mm optical zone, and the near correction over a 6.5 mm optical zone; the ring between the 5 and 6.5-mm optical zone provides the multifocality [21]. It improves near vision by creating a prolate corneal shape with negative spherical aberration to increase depth of field [22].

Pinelli et al. reported the results using the PML technique in 44 hyperopic eyes, mean binocular UCVA was 1.06 ± 0.13 for distance and 0.84 ± 0.14 for near. 4.5% of the eyes lost 1 line of CDVA, and 45% of eyes gained 1 line of CDVA. They also reported a reduction in contrast sensitivity and a decrease in spherical and an increase in coma aberration [21].

Gordon reported a follow up of 3 months of 102 patients using the PML technique, and 81% of the patients had 20/20 UDVA, 44% had J1, 60% had J2, and 96% had a J3 UNVA. There was no loss of UDVA neither were there any visual complains [22] .

Epstein et al. investigated the outcomes of combination of monocular peripheral presbyLASIK on the non-dominant eye and monofocal distance vision correction on the dominant eye; the study included 103 patients (myopes and hyperopes) with a follow up of 1.1 to 3.9 years. 91.3% of all patients reported complete spectacle independence (89% hyperopes and 92% of myopes), UDVA was at least 20/20 in 67.9% of hyperopes and 70.7% of myopes. Seventy-one percent of hyperopes and 65.3% of myopes had a 20/20 vision at 40 cm; 14.3% of hyperopes lost one line of CDVA. There was no significant change in stereoacuity. Spherical aberration increased in the myopic group but decreased in the hyperopic group. All eyes that had PresbyLASIK had a statistically significant increase in total HOAs [23].

Danasoury et al. reported the outcomes of peripheral presbyLASIK in hyperopes and myopes with a follow up of 1 year. For the treatment of hyperopia and presbyopia a hyperopic ablation was performed with a 7.0 mm optical zone and a 9.5 mm transitional zone, the induced myopia due to the presbyopic correction was reversed centrally using two consecutive myopic ablations with optical zones of 3.5 and 4.0 mm with a transition zone that was 1.0 mm larger than the respective optical zones.

The treatment of the myopic group involved an ablation using 2 or 3 concentric optical zones at 4.0, 5.0, and 6.0 mm with a 2 mm transition zone that was larger than the optical zone. Presbyopia was treated with a hyperopic ablation with an optical zone of 7.00 and a 9.5 mm transition zone. The induced myopia was then reversed. Distance UCVA in the hyperopic group was 20/40 or better in 94% of the eyes, 20/25 in 83% and 20/20 or better in 56% of the eyes. In the myopic group, 44% of the eyes had UDVA of 20/20, 78% had 20/25 or better, and 90% had 20/40 or better.

Thirty-three percent of hyperopes had 20/40 or better UNVA and 36% of the myopes; even though myopes had a better UNVA than hyperopes, were least satisfied by the results (48% of myopes were satisfied *vs*. 54% of hyperopes). Two percent of eyes in each group lost two lines of CDVA. In the hyperopic group, there was a statistically significant change in ocular and corneal spherical aberration, but this was not observed in the myopic group [24].

See Table 2 for a review of peripheral PresbyLASIK results.

**Table 2:** Published outcomes for presbyopia correction with Peripheral PresbyLASIK

Author	Procedure	Follow up	No. of patients	Median Age (years)	UNVA	UDVA	Safety	Spectacle independence / Satisfaction	Retreatments
Pinelli et al. [21]	PML	6 months	22 hyperopes	56	0.84 ± 0.14	1.06 ± 0.13	4.5% lost 1 line of BSCVA	82% were very satisfied	12% improvement of distance vision
Gordon et al. [22]	PML	3 months	102 hyperopes and myopes	>40	44%→ J1, 60% →J2	81%→ 20/20	No visual loss	NA	10% higher than standard LASIK treatment (typically ~2%)
Epstein et al. [23]	Peripheral presbyLASIK in the non-dominant eye, monofocal LASIK in the dominant eye	1 year	103 hyperopes and myopes	53.3	71.4% hyperopes and 65.3% myopes had 20/20 at 40 cm	67.9% hyperopes →20/20. 70.7% myopes →20/20	14.3% hyperopes lost 1 line of distance BSCVA	91.3% had spectacle independence. 89% hyperopes, 92% myopes	26.6% of myopes. 28.6% of hyperopes.
Danasory et al. [24]	Peripheral presbyLASIK	1 year	34 Hyperopes and 39 myopes	49 ± 5.6	56% hyperopes and 44% of myopes had 20/20 or better	60% of the hyperopes →J2 84% of the myopes→J2	2% of each group lost 2 lines of BSCVA	58.9% of the hyperopes and 55.5% of the myopes were not using spectacles for reading the newspaper.	18.75% of the hyperopes and 28% of the myopes were retreated

*NA=* Information not available, *PML=* peripheral multifocal LASIK, *UNVA=* uncorrected near visual acuity, *UDVA=* uncorrected distance visual acuity, *BSCVA=* best spectacle-corrected acuity

### Laser Blended Vision (LBV, Carl Zeiss Meditec)

This technique induces a controlled spherical aberration (to increase depth of field [25], the induced negative spherical aberration goes from -0.50 to -0.70 μm) within a limited range to avoid degradation of the visual quality, with a small degree of monovision [26] to provide good near and distance vision. It can be performed on emmetropic, myopic and hyperopic presbyopes.

Reinstein et al. [25] reported the outcomes of LBV on emmetropic presbyopes; 96% had an UNVA of J2, the same outcomes were achieved for the treatment of myopic astigmatism and presbyopia [27]. In the case of hyperopic presbyopes, 81% of patients achieved a UNVA of J3 [28].

Yin et al. [7] used central presbyLASIK in the dominant eye and Q factor modulation (increase in negative Q factor for the improvement of depth of focus) in the non-dominant eye; the study included only hyperopes. The mean UNVA achieved was Jaeger 2, a mean UIVA and UDVA of 20/20. Regarding safety, 1 eye lost 2 lines and 5 eyes lost 1 line of CDVA one month after surgery, even though, 100% of the patients were satisfied with their results at 1 year after surgery.

Vastardis et al. [29] reported the outcomes of a multifocal aspheric corneal ablation, two groups were created, in one the target was emmetropia, and in the other group the target was a slight myopia (-0.5 D). In both groups, there was a significant improvement in UNVA, UIVA and UDVA and mini-monovision did not seem to affect the UDVA, UIVA and UNVA. A significant loss of lines of CDVA in both groups occurred.

A 6 months follow up was reported by Courtin et al. [30]. They used the Custom-Q nomogram (Alcon Laboratories, Inc., Fort Worth, TX), which allows the surgeon to select a target refraction and a target corneal asphericity. Only presbyopic hyperopes were included in the study. In the non-dominant eye, an aspheric ablation profile was planned, associated with a myopic refraction. A binocular UDVA of 20/20 was achieved in 91% of the patients, with 83% having a Jaeger 1 or better binocular UNVA.

See Table 3 for LBV results.

**Table 3:** Published outcomes for presbyopia correction with Laser Blended Vision

Author	Procedure	Follow up	No. of patients	Median Age (years)	UNVA	UDVA	Safety	Spectacle independence/Satisfaction	Retreatments
Reinstein et al. [25]	Non-linear Aspheric micro-﻿monovision. Target refraction was plano in the dominant eye and between -1.00 and -1.88 diopters in the non dominant eye.	1 year	148 emmetropes	55	96% → J2	95% → 20/20	From the eyes that lost 1 line, 99.3% achieved CDVA of 20/20	NA	11.8%: 40% for distance and 60% for near.
Reinstein et al. [27]	Non- linear Aspheric myopic micro-monovision. Target refraction was plano for the dominant eye and between -0.75 and -2.00 diopters in the non dominant eye.	1 year	155 myopes with astigmatism	49	96% → J2	99% → 20/20	22 eyes lost 1 lineof UDVA	NA	19%: 52% for distance and 48% for near
Reinstein et al. [28]	Non-linear aspheric profile with -1.5 diopters of micro-monovision in the non-dominant eye.	1 year	111 hyperopes	56	81% → J2	99% > 20/25	17% lost 1 line of CDVA	NA	22%: 50% for distance, 50% for near
Yin et al. [7]	Central PresbyLASIK with corneal asphericity modulation in the non-dominant eye	1 year	69 hyperopes	53.84 ± 4.19	70% → J2	100% → 20/20	1.22% lost 2 lines, 6% lost 1 line of CDVA	100% of patients were satisfied.	16 patients, 7 in the non-dominant eye, 7 in the dominant eye, 2 bilateral retreatments
Courtain et al. [30]	Dominant eye plano target refraction, non-dominant eye aspheric ablation profile and a myopic shift.	6 months	49 hyperopes	56.5 ± 5.7	83% → J1	91% → 20/20	1 patient lost 1 line of CDVA	NA	Re-treatment rate was 10.8%. 5 patients in the non-dominant eye, 2 patients in the dominant eye.

*UNVA=* uncorrected near visual acuity, *UDVA=* uncorrected distance visual acuity, *CDVA=* corrected distance visual acuity

## Conclusions

With this review, we can conclude that almost all authors reported a loss of at least 2 lines of distance visual acuity [8, 10, 11, 16, 18, 19, 21, 23, 24], which is a highly undesirable risk. The loss of vision can be secondary to dry eye or the induction of HOAs [11]. Patient selection seems crucial for having good results [11, 13]; the surgeon has to take into account patient expectations, their jobs and hobbies to see if they are good candidates for the procedure.

Most of the PresbyLASIK treatments have been performed in hyperopic patients [4, 7, 8, 10–12, 14]. These patients are more satisfied with their outcomes than myopes [24], since the latter have always been used to having good near vision.

Different techniques of presbyLASIK are available (central, peripheral, blended vision) however, there is much more scientific evidence with the use of central presbyLASIK technique [7, 8, 10–12, 14, 16–19, 29] than with the use of peripheral presbyLASIK [21–24]. Peripheral presbyLASIK removes an important amount of corneal tissue especially in the myopes, making this a limitation of the technique [6]. Central presbyLASIK can be performed in either myopes or hyperopes as the amount of tissue necessary for removal is minimal. Furthermore, the central model is more advisable to achieve multifocality due to the physiologic pupil miosis during accommodation [31].

One of the main limitations of PresbyLASIK is the lack of strong scientific evidence, and there are no reports of long term follow-ups, most of the papers have only a 6 to 12 months follow-up [7, 8, 10, 11, 21–23, 29]. Spectacle independence varies from 72% [8] to 93% [12].

The combination of induced asphericity and micro-monovision with laser blended technique has had good visual and safety outcomes [25, 27–30], but the tolerance to micro-monovision may be inconvenient especially in patients with mild presbyopia, who are less tolerant to a larger degree of anisometropia than patients with advanced presbyopia [25].

Presbyopia correction at the cornea can also be achieved with monovision, in which an intended anisometropia is induced, usually, the non-dominant eye is corrected for near vision, and the dominant eye for far vision, it depends on inter-ocular blur suppression. Good visual outcomes are achieved with this technique [32], but there is a loss of stereopsis which is related to the degree of anisometropia [33, 34], it is generally contraindicated in patients that need a good stereopsis to perform their daily activities such as airplane pilots [35, 36] or professional drivers [33, 36].

Corneal inlays are other way for the correction of presbyopia at a corneal level, depending on the inlay, they can either provide a multifocal effect by creating a hyper-prolate region of increased power in the cornea; improve the depth of focus, or they can act by altering the refractive index with a bifocal optic [37]. One of the advantages of corneal inlays over monovision or presbyLASIK is that there is no need of corneal tissue ablation, but, the patient must tolerate monovision and a loss of distance vision has been reported [38–42].

Most of the procedures for presbyopic correction at a corneal level have the risk of losing lines of distance vision, but other procedures like the implantation of multifocal IOL in cataract surgery also carries risks like endophthalmitis, macular edema, suprachoroidal hemorrhage or retinal detachment [43].

Achieving a multifocal cornea with stable and long term results remains a challenge [7, 11, 13, 44] to all refractive surgeons. The combination of different techniques for the correction of presbyopia (monovision, multifocality, asphericity modification) is a trending option [30] seeing that they benefit from the best qualities of each procedure.

## Abbreviations

CDVA: Corrected distance visual acuity
CNVA: Corrected near visual acuity
UDVA: Uncorrected distance visual acuity
UIVA: Uncorrected intermediate visual acuity
UNVA: Uncorrected near visual acuity.
